# Case report: Envafolimab combined with Endostar in the treatment of advanced non-small cell lung cancer with malignant pleural effusion

**DOI:** 10.3389/fonc.2024.1368059

**Published:** 2024-04-04

**Authors:** Changhong Dong, Chenxi Hu, Yanting Jiang, Kaiyuan Hui, Xiaodong Jiang

**Affiliations:** Department of Oncology, The Affiliated Lianyungang Hospital of Xuzhou Medical University, Lianyungang, Jiangsu, China

**Keywords:** non-small cell lung cancer, malignant pleural effusion, immune checkpoint inhibitors, anti-angiogenic therapy, envafolimab, Endostar, case report

## Abstract

Malignant pleural effusion (MPE) is one of the common complications of lung cancer. The quality of life and prognoses for MPE patients are significantly compromised. Controlling the production of MPE can relieve patients’ symptoms, improve their quality of life, and prolong their survival. This article presents a case of advanced non-small cell lung cancer (NSCLC) with MPE and negative driver genes. The patient received envafolimab and Endostar in combination, resulting in a complete reduction of MPE and durable clinical benefits. The exploratory use of this treatment method improved the quality of life of this patient and has the potential to prolong the survival of this patient.

## Introduction

Lung cancer is the second-most commonly diagnosed cancer and the leading cause of cancer death worldwide (1). Among all the malignant tumors, malignant pleural effusion (MPE) is a common complication, with the greatest incidence found in patients with lung cancer (2). The quality of life and prognoses for MPE patients are significantly compromised (3). Controlling the production of MPE can relieve patients’ symptoms, improve their quality of life, and prolong their survival (4). However, for patients with lung cancer accompanied by MPE, there is currently no recommended therapeutic strategy. Currently, there is an urgent need for an efficient and standardized treatment strategy.

MPE is rich in cytokines and chemokines, such as interleukin, transforming growth factor, and vascular endothelial growth factor (VEGF) (5, 6). Multiple treatment strategies for MPE have focused on targeting VEGF (6, 7). In addition, the pleural space of MPE is considered to be a tumor-tolerant environment (8). However, immunotherapy may stimulate tumor-specific immune responses in the pleural space to reverse tumor-tolerant environment (8). Therefore, immunotherapy has been a particularly interesting area of treatment for MPE (8).

In recent years, immune checkpoint inhibitors (ICIs), mainly targeting programmed death 1 (PD-1) and programmed death-ligand 1 (PD-L1), have transformed the treatment paradigm of advanced non-small cell lung cancer (NSCLC) and become an important component of NSCLC therapy (9). Due to the improved efficacy and reduced adverse effects of the combination of anti-angiogenic therapy and ICIs, this combined therapeutic approach has also become one of the main research directions. However, phase III trial data are scarce regarding the impact of the combination of ICI and anti-angiogenic therapy on NSCLC with MPE.

Endostar is an anti-angiogenic agent developed in China that has been recommended as a first-line treatment option for NSCLC (10). In patients with NSCLC accompanied by MPE, Endostar can significantly improve the control rate of MPE and reduce its recurrence rate (11). Envafolimab, a novel subcutaneous single-domain anti-PD-L1 antibody, has been approved for treating microsatellite instability-high (MSI-H) or DNA-mismatch repair (dMMR) solid tumors in China. We found that there are few studies on the use of Endostar and envafolimab in the treatment of MPE in NSCLC. This article presents a case of advanced lung adenocarcinoma with MPE that received first-line treatment with envafolimab combined with Endostar.

## Case presentation

**Figure 1:**
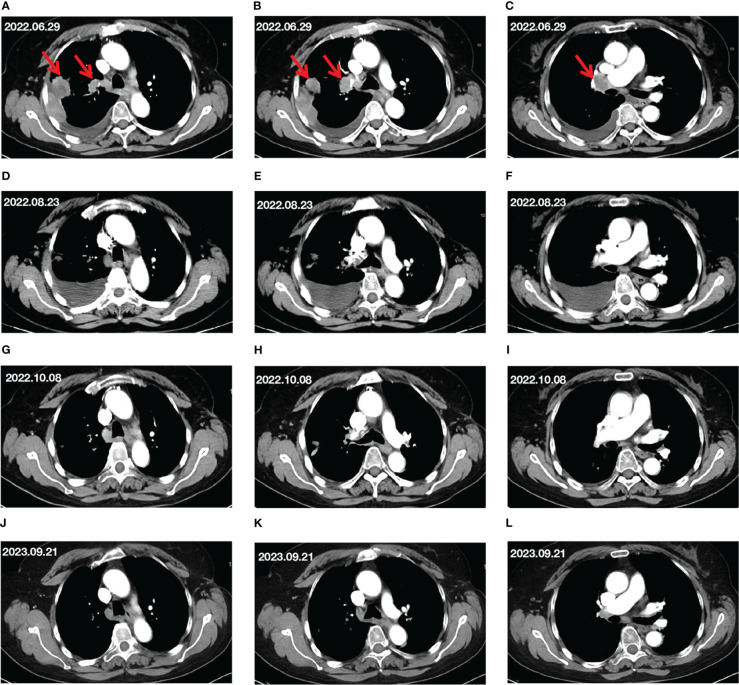
Changes in target lesion during treatment. **(A–C)** CT scan before treatment. **(D–F)** CT scan after 2 cycles of treatment. **(G–I)** CT scan after 4 cycles of treatment. **(J–L)** CT scan after 15 months of treatment. The red arrows mark the target lesions.

This report concerns a 72-year-old female patient who presented with chest and back pain. Ahead of hospitalization, the patient had stable vital signs, and the Eastern Cooperative Oncology Group (ECOG) performance status (PS) score was 1 point. Computed tomography (CT) imaging revealed a right lung mass invading the adjacent pleura, enlarged right hilar lymph nodes, a small amount of right pleural effusion, and no abnormalities in the head (**Figures 1A–C**). According to the tumor marker test results, the level of cytokeratin-19 fragment (CYFRA21-1) was 11.061 ng/mL, and the level of carcinoembryonic antigen (CEA) was 35.08 ng/mL, indicating an upward trend in tumor markers. A percutaneous lung biopsy was performed, and the pathological results revealed adenocarcinoma. The final diagnosis was right lung adenocarcinoma of stage IV (T_2_N_1_M_1_). Driver gene and PD-L1 testing were performed by analyzing tissue samples, which showed no mutations in epidermal growth factor receptor, anaplastic lymphoma kinase, or *C-ROS* oncogene 1, together with a PD-L1 tumor proportion score (TPS) of 70%.

The patient refused chemotherapy and ultimately received immunotherapy combined with anti-angiogenic therapy. On July 11 and August 1, 2022, the patient received two cycles of treatment with envafolimab (300 mg, d1) combined with Endostar (210 mg, d1–3) every 3 weeks.

After two cycles of treatment, the patient’s chest and back pain were reduced somewhat. CT imaging on August 23, 2022, showed that the right lung lesion had shrunk but the right pleural effusion had increased (**Figures 1D–F**) compared to the CT results before treatment. Besides, the tumor markers included the following results: CEA at 10.88 ng/mL, CA-199 at 94.32 U/mL, and CYFRA21-1 at 3.62 ng/mL. Although the lung lesions and tumor marker levels had decreased, the patient’s right-side pleural effusion had increased, and the patient presented with symptoms of chest tightness. Therefore, based on the original plan, thoracentesis was performed to drain the pleural effusion. The patient received envafolimab combined with Endostar for the third and fourth cycles of treatment on August 23 and September 14, 2022.

After three cycles of treatment, the patient’s chest tightness and chest and back pain were alleviated.

After completing four cycles of treatment, a CT scan on October 8 showed that the right lung lesions and right hilar lymph nodes had slightly shrunk, and the pleural effusion was also decreased (**Figures 1G–I**) compared to its size on August 23, 2022. At this point, the tumor-marker results included the following: CEA at 2.11 ng/mL, and CYFRA21-1 at 3.77 ng/mL. The imaging evaluation of efficacy was a partial response (PR) according to the Response Evaluation Criteria in Solid Tumors guidelines, and the ECOG PS score was 0 points.

From October 8, 2022, to November 11, 2023, the patient received combined treatment with envafolimab and Endostar for the fifth cycle through to the 22nd cycle. The CT evaluation on September 23, 2023 showed that the lung lesions were stable, and the MPE had disappeared (**Figures 1J–L**). The evaluation of efficacy was PR, and the tumor markers were basically within normal ranges (CEA, 1.75 ng/mL; CYFRA21-1, 2.13 ng/mL). The patient’s ECOG PS score was 0 points.

It is worth noting that the main adverse event recorded during treatment was hypothyroidism. According to the National Cancer Institute Common Toxicity Criteria (NCI-CTC AE, Version 5.0), the adverse event was evaluated as grade 2. Following oral administration of levothyroxine tablets, the patient’s thyroid function returned to her normal range (TSH, 2.95 mIU/L; FT3, 5.26pmol/L; FT4, 10.01 pmol/L; T3, 2.07nmol/L; T4, 122.33 nmol/L). The patient received first-line treatment with envafolimab combined with Endostar and has not shown disease progression so far. To date, the progression-free survival (PFS) of this patient has reached 15.9 months.

## Discussion

In this case, our patient was diagnosed with advanced lung adenocarcinoma accompanied by MPE. Notably, her driver gene test result was negative, and her PD-L1 expression was high (TPS=70%). After 22 cycles of envafolimab combined with Endostar treatment, the patient’s right lung lesions and hilar lymph nodes had significantly shrunk, and her MPE had also completely disappeared without recurrence. Overall, the patient achieved durable clinical benefits. During treatment, the main adverse event was hypothyroidism, which was evaluated as grade 2, indicating the good safety of this regimen.

Currently, there is no recommended standard treatment strategy for NSCLC patients with MPE. Considering her advanced age and refusal to receive chemotherapy, the patient ultimately decided to receive immunotherapy combined with anti-angiogenesis therapy, which involved the use of envafolimab in combination with Endostar. After two cycles of treatment, the increase in MPE in the patient may be due to several factors. First, after receiving anti-tumor treatment, the lesion in the pleura was significantly reduced, which may lead to fluid leakage of tumor vessels, thus promoting the increased production of MPE. Secondly, immune cells may exacerbate lymphatic drainage obstruction during the clearance of tumor cells, resulting in lymphatic reflux obstruction. Finally, immune cells may trigger local inflammatory reactions and enhance capillary permeability during the process of killing tumor cells, which may all promote the increase in MPE (12).

There is a theoretical basis for the combined application of anti-angiogenic therapy and ICIs. VEGF, a crucial inducer of tumor angiogenesis, regulates immune responses (13). It reduces T-cell infiltration and activity by downregulating adhesion molecules and enhancing PD-1 expression (14). VEGF also inhibits dendritic cell maturation and activation, limiting antigen presentation (15, 16). VEGF prompts secretion of prostaglandin E2, promoting suppressor cell infiltration and regulatory T cell differentiation (17, 18). VEGF induces Fas ligand expression on tumor endothelial cells to mediate apoptosis of effector T cells (14). Therefore, anti-angiogenic can improve the immunosuppressive microenvironment of tumors.

Currently, many clinical trials have investigated the efficacy of combination therapy of anti-angiogenesis and ICIs in NSCLC. Seto et al. evaluated the efficacy of bevacizumab combined with atezolizumab as the first-line treatment for advanced non-squamous NSCLC with driver gene negativity and high PD-L1 expression (19). In all subjects, the objective response rate (ORR) reached 42.9%, the median PFS was 15.9 months, and no treatment-related adverse effect of grade 4 or 5 occurred (19). The previous Impower-110 study evaluated the efficacy of atezolizumab monotherapy in the first-line treatment of advanced NSCLC with driver gene negativity and high PD-L1 expression (20). Impower-110 study showed that advanced NSCLC patients treated with atezolizumab monotherapy had an ORR and median PFS of 38.3% and 8.1 months, respectively (20). The subject populations in these two studies were similar, both consisting of previously untreated patients with advanced NSCLC who were negative for driver genes and had high PD-L1 expression. The study by Seto et al. (19) suggests that bevacizumab combined with atezolizumab has a satisfactory efficacy. However, further large randomized controlled studies are needed to validate the efficacy and safety of their combination.

MPE is considered an immunological and vascular manifestation of malignant pleural metastasis (21, 22). Inhibitors that block VEGF activity are thought to effectively manage MPE (23). With the promise of ICIs in lung cancer and other malignancies, there has been a renewed interest in investigating the efficacy of local and systemic ICIs for patients with MPE (23, 24). However, ICI monotherapy for the treatment of MPE is currently not recommended, possibly due to the increased mortality associated with MPE. In 2019, a retrospective study evaluating the efficacy of advanced NSCLC patients treated with ICI to verify whether ICI monotherapy is suitable in the presence of MPE and found that ICI monotherapy did not improve survival outcomes in patients with MPE (25). In 2022, a multicenter retrospective study evaluated the efficacy in NSCLC patients with MPE who received either pembrolizumab monotherapy or ICI combined with chemotherapy (26). Compared with ICI monotherapy, the PFS in the ICI combined with chemotherapy group increased significantly (11.1 vs 3.9 months, p=0.04) (26). Therefore, ICI monotherapy may not be appropriate for NSCLC patients in the presence of MPE.

However, there is currently a dearth of data about the response of NSCLC patients with MPE to the combined therapy of ICIs and anti-angiogenic therapy.

The successful treatment of this case provides strong evidence support for the application of envafolimab combined with Endostar in advanced lung adenocarcinoma with MPE. In the future, we look forward to further validating the efficacy and safety of this treatment regimen in advanced NSCLC through more clinical studies.

## Data availability statement

The original contributions presented in the study are included in the article/**Supplementary Material**. Further inquiries can be directed to the corresponding authors.

## Ethics statement

Written informed consent was obtained from the individual(s) for the publication of any potentially identifiable images or data included in this article.

## Author contributions

CD: Writing – review & editing, Data curation, Funding acquisition, Writing – original draft. CH: Writing – original draft, Conceptualization, Investigation, Software, Visualization. YJ: Writing – original draft, Project administration, Supervision, Validation. KH: Project administration, Supervision, Validation, Writing – review & editing. XJ: Project administration, Supervision, Validation, Writing – review & editing.
