# Supplementary material for: Case report: Envafolimab combined with Endostar in the treatment of advanced non-small cell lung cancer with malignant pleural effusion
Source: Front Oncol. 2024 Apr 4;14:1368059. doi: 10.3389/fonc.2024.1368059 (PMC11024318; doi:10.3389/fonc.2024.1368059)
Supplement: Supplementary file 1 [file DataSheet_1.pdf]

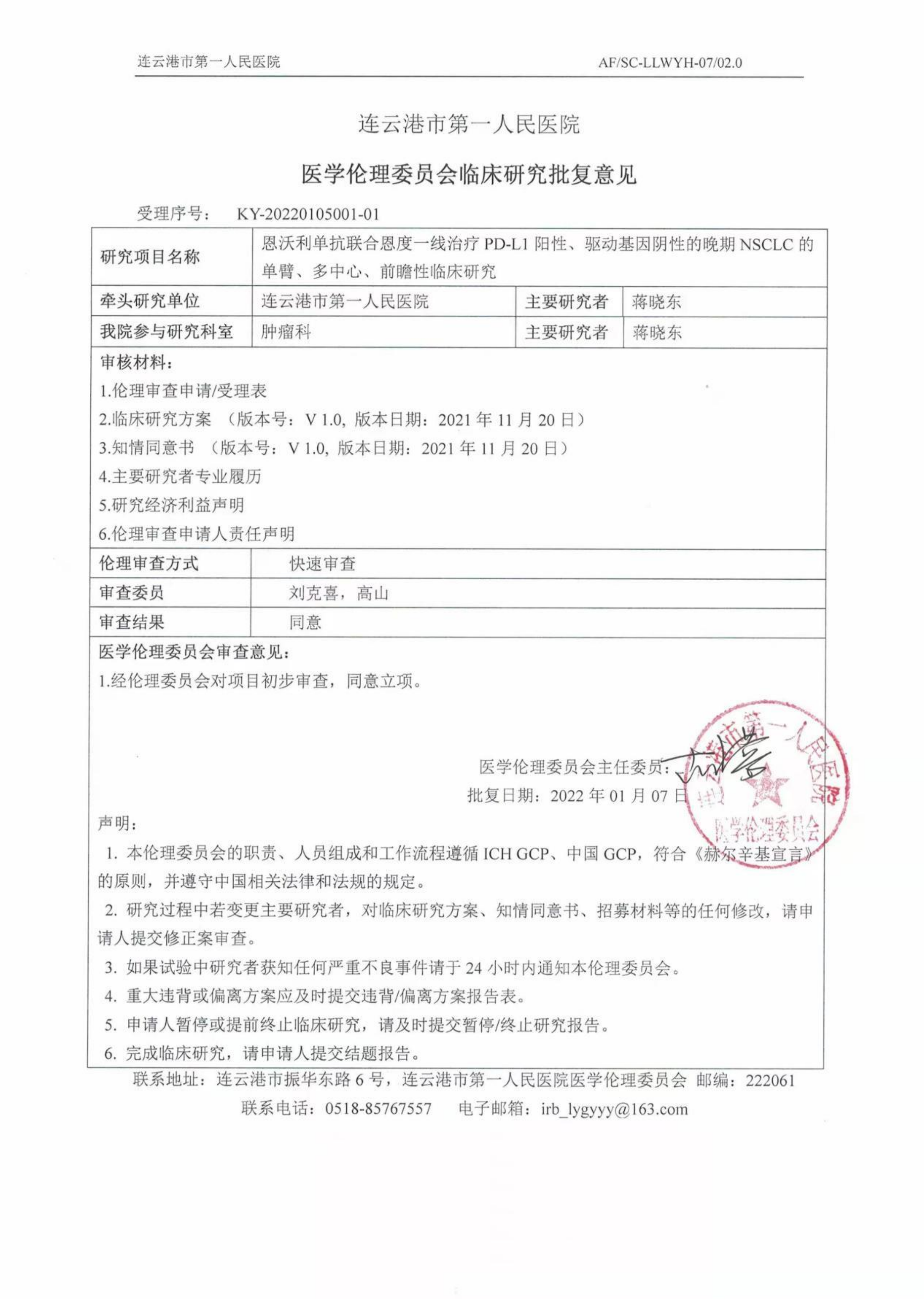

连云港市第一人民医院　　AF/SC-LLWYH-07/02.0

连云港市第一人民医院

医学伦理委员会临床研究批复意见

受理序号：　KY-20220105001-01

**研究项目名称**	恩沃利单抗联合恩度一线治疗 PD-L1 阳性、驱动基因阴性的晚期 NSCLC 的单臂、多中心、前瞻性临床研究		
**牵头研究单位**	连云港市第一人民医院	**主要研究者**	蒋晓东
**我院参与研究科室**	肿瘤科	**主要研究者**	蒋晓东

审核材料：

1.伦理审查申请/受理表

2.临床研究方案 （版本号：V 1.0，版本日期：2021 年 11 月 20 日）

3.知情同意书 （版本号：V 1.0，版本日期：2021 年 11 月 20 日）

4.主要研究者专业履历

5.研究经济利益声明

6.伦理审查申请人责任声明

**伦理审查方式**	快速审查
**审查委员**	刘克喜，高山
**审查结果**	同意

医学伦理委员会审查意见：

1.经伦理委员会对项目初步审查，同意立项。

医学伦理委员会主任委员：

批复日期：2022 年 01 月 07 日

声明：

1. 本伦理委员会的职责、人员组成和工作流程遵循 ICH GCP、中国 GCP，符合《赫尔辛基宣言》的原则，并遵守中国相关法律和法规的规定。
2. 研究过程中若变更主要研究者，对临床研究方案、知情同意书、招募材料等的任何修改，请申请人提交修正案审查。
3. 如果试验中研究者获知任何严重不良事件请于 24 小时内通知本伦理委员会。
4. 重大违背或偏离方案应及时提交违背/偏离方案报告表。
5. 申请人暂停或提前终止临床研究，请及时提交暂停/终止研究报告。
6. 完成临床研究，请申请人提交结题报告。

联系地址：连云港市振华东路 6 号，连云港市第一人民医院医学伦理委员会 邮编：222061

联系电话：0518-85767557　　电子邮箱：irb_lygyyy@163.com

sFigure 1. Ethical Approval Document from the Ethics Committee

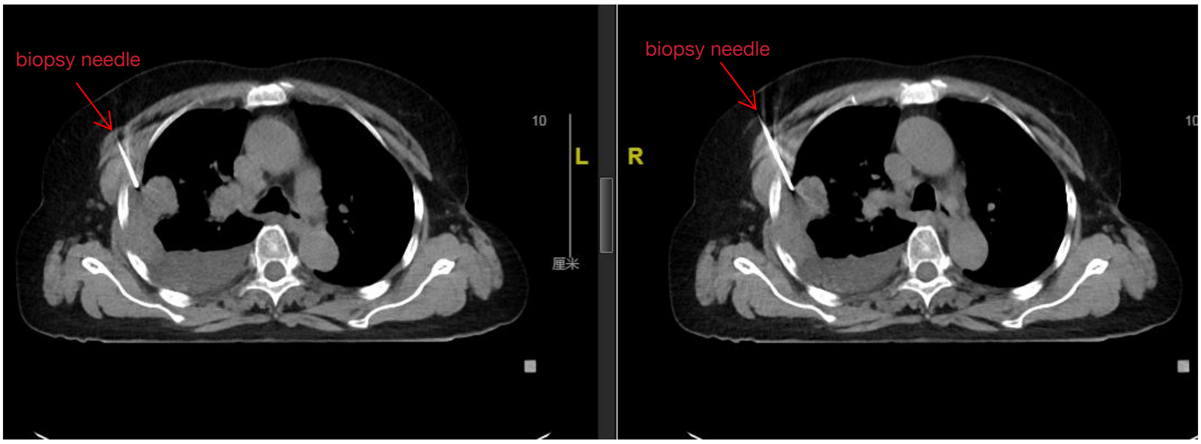

sFigure 2: The image of CT-guided tissue biopsy

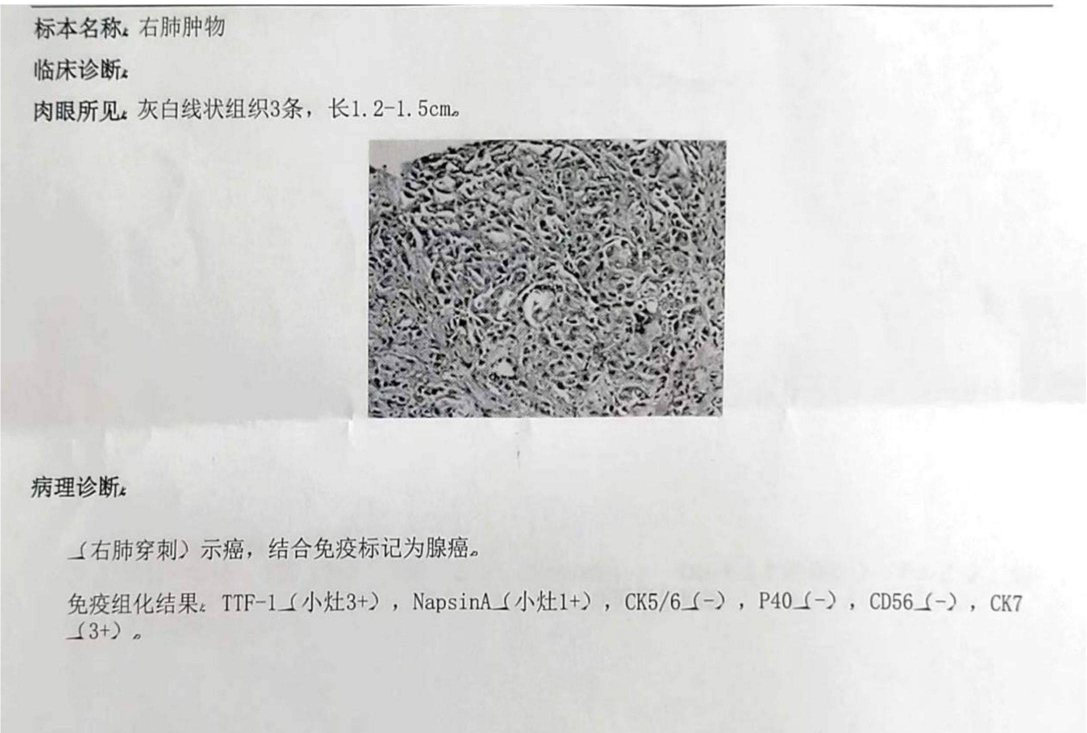

标本名称：右肺肿物

临床诊断：

肉眼所见：灰白线状组织3条，长1.2-1.5cm。

病理诊断：

（右肺穿刺）示癌，结合免疫标记为腺癌。

免疫组化结果：TTF-1（小灶3+），NapsinA（小灶1+），CK5/6（-），P40（-），CD56（-），CK7（3+）。

sFigure 3. Pathological report of this patient
